# Factors influencing the mutual-support willingness and needs among the rural elderly in Hunan Province, China: a cross-sectional study

**DOI:** 10.1186/s12913-022-07677-0

**Published:** 2022-03-05

**Authors:** Ke-ru Yao, Xin-hong Yin, Qin Luo, Xi Tang, Xiu-zhu Tan

**Affiliations:** School of Nursing, University of South China, Hengyang, 421000 China

**Keywords:** Elderly, Rural district, Mutual-support, Eldercare, Social support

## Abstract

**Background:**

This study aimed to assess the influence factors of the mutual-support willingness and identify the mutual-support needs of elderly living in rural areas of Hunan Province, China.

**Methods:**

Using the Chi-square test and logistic regression to analyze factors influencing Participants’ mutual-support willingness and needs.

**Results:**

Factors influencing the mutual-support willingness and needs included individual characteristics, family environment, and so on. And the rural elderly’s demand for mutual-support is at a relatively high level. The total score for social support for the aged was 36.944 ± 6.487, at a moderate level.

**Conclusions:**

It is necessary to objectively evaluate the factors related to mutual-support willingness and needs and take steps to enhance social support and meet elderly the needs of mutual-support, which is of great significance for improving the happiness of the elderly in their later years and alleviating the crisis of population aging in China.

## Background

China has the world’s largest elderly population. At the end of 2019, the number of elderly people aged 60 and over in China reached 254 million, accounting for 18.1% of the total population [1]. The report forecasts that in 2050, there will be approximately 483million people aged 60 and over in China, accounting for about 34.1% of the total population [2]. A large population base and rapid development are typical characteristics of China’s population aging. The continuous growth of the aging population has increased the burden of old-age care in China. Among all the elderly, rural elderly account for more than half. Due to the acceleration of our country’s urbanization and the transfer of young and middle-aged rural labor, the power of migration to cities has made the rural population age faster than urban cities, and rural areas are also facing serious problems of aging [3]. For rural communities, with the development of the economy and society, the general situation is that the villages are hollowed out, the population is aging, households are empty, and lives are impoverished [4]. Thus, the eldercare needs of older adults will grow substantially. Collectively, it is obvious that there is a clear challenge to meeting the care and support needs of an aging population both now and in the future. Whereas assistance from family members may be limited, assistance from others or neighbors is more accessible and thus becomes more important [5, 6].

Because of the expansion of eldercare needs and the shortage of eldercare teams, the mutual-support model was developed by learning from foreign “time banks” and combining it with China’s national conditions. The time banks was first proposed by Edgar S. Cahn in 1986 [7]. The time banks mean a new model for volunteering. The essence of this model is to take the time bank as the intermediary to achieve the deferred payment of labor achievements by integrating community resources and quantifying service time, to achieve the purpose of mutual-support in the community. Examples include the “Village” model in American. The “Village” model mainly relies on membership dues to support the operation of the organization. Members also act as managers and volunteers for the “village” model. Through mutual support services, volunteers meet the elderly care needs of members [8]. The “Village” model helps the elderly obtain needed health and social services to increase their ability to age in place [9]. The model of living together and cooperating with the elderly in the UK depends on the participation of the housing association, whose members are people aged 50 and over. The main purpose of this model is not to meet the material needs of the elderly, but to focus on life-care and spiritual aspects in order to maintain a positive living state through living together [10]. “Multi-generation residence” is the feature of the German mutual-support model, which aims to gather people from different families and ages and live in the same community. Young people mainly alleviate the loneliness of the elderly through daily communication or by providing life-care for the elderly by doing trivial things. The elderly also provide free housing for young people to ease the economic pressure of young people [11]. One study from 17 countries from the Survey of Health, Ageing, and Retirement in Europe (SHARE) suggests that the provision and receipt of help among old adults are driven by personal characteristics, health resources, living situation, social factors, and contextual factors [12]. And a comprehensive study selected individuals, family environment, and community environment as potential factors which may affect the willingness to accept elder care [13]. The “neighborhood mutual-support network” in Japan is primarily for urban elderly people aged 65 and up who live alone or are widowed. With the support of local government materials, policies, and volunteers (local middle-aged and elderly residents, especially housewives and retirees), they formed a stable association mutual-support group [14]. A Japanese study shows that 57.8% of the elderly in rural areas have life support intentions for others, and the intention of the undertaker of life support is generated according to the needs of the recipient of life support [15]. The description of these personal and mutual-support needs is scarce in previous literature. Therefore, it is crucial to investigate the determinates of given and received help among older adults [16]. One study showed high reciprocity of neighbors among older adults. However, reciprocity also implies that older adults should not be viewed only as recipients but also as active providers of assistance to others in their neighborhood. Indeed, active helpers often filled gaps in social services in the neighborhood. Moreover, the intervention to promote social exchange in a neighborhood should focus on the individual’s life course, social networks, and country characteristics [17].

Previous Chinese studies investigated the factors influencing the willingness to receive mutual-support among the elderly in the Chinese population [18–20]. Socioeconomic and demographic factors, including age, marital status, economic status, and so on, are associated with the willingness to receive mutual-support [18, 20]. Community support significantly improves villagers’ willingness to participate in mutual-support for the aged [19]. A study conducted on 315 retired elderly living in Shanghai City showed that gender, age, education level, number of children, and physical health status are associated with the elderly’s intention to receive mutual-support [21]. And a study conducted in the rural area of a poverty-stricken county in Hunan province showed that the factors that influence the willingness to receive mutual-support for empty-nest elderly are age, education, self-care agency level, number of chronic diseases, and health promotion lifestyle [22]. Mutual-support needs and social support were important factors influencing the elderly’s willingness to receive mutual-support. However, past researchers have not reported the effect of mutual-support needs and social support on the elderly’s mutual-support willingness.

To our knowledge, no studies to date have investigated the factors influencing the mutual-support willingness and needs among the rural elderly in China. In light of the Chinese aging problem, our study focused on the rural elderly in Hunan Province, China, and explored the factors influencing mutual-support willingness and needs as well as the correlation of mutual-support willingness with social support. The results of this study provide reference information for informing the development of social eldercare and health promotion programs, improving the quality of aging services. What’s more, that can contribute to the more rational allocation of eldercare resources and satisfy the rural elderly’s eldercare needs.

## Methods

### Survey methods

Rural elderly: people over the age of 60 who have a registered address in agriculture and live in a rural area. This was a cross-sectional study. The study used a convenience sampling method. The participants were 2167 elderly people aged 60 years or older from Hunan province in China. The study was conducted from September 2020 to March 2021.

According to urban planning, the regional economy of Hunan Province is divided into four regions. For the four districts, we selected Changsha, Yueyang, Huaihua, and Hengyang for questionnaire distribution. We contacted and cooperated with township medical personnel in four regions in advance. Each of township health center in different regions will have a person in charge. The electronic questionnaire was distributed to the potential participants by Wechat (Free applications for instant messaging services for smart terminals) promotion of responsible people in different regions. All investigators had received consistent training before the investigation. Those who agreed to participate in the survey can fill it online. The elderly who are illiterate or difficult to fill in will be assisted by volunteers or their families. And the respondents’ answers were collected by Questionnaire star (a website for online questionnaire filling and collection) after they had completed.

### Measurements

The study’s instrument was a self-administered questionnaire that consisted of three sections. Section 1 consisted of the general information questionnaire, which items included individual characteristics, family environment, economic status, and medical factors. Section 2 measured the mutual-support needs of respondents, items corresponding to daily life care service needs, health service needs, spiritual comfort service needs, entertainment and learning needs. The question and answer for each item shall be scored by Likert’s five grades. It consists of 20 items with a total score ranging from 20 to 100; a higher score indicates a higher level of mutual-support needs. A score of it ≤34 is considered low mutual-support needs, one between 34 and 66 is moderate mutual-support needs, and one of it ≥66 indicates high mutual-support needs. Section 3 was the social support rating scale (SSRS), which was established in 1986 by Xiao Shuiyuan. In this study, the social support rating scale was used to measure the social support of the participants [23]. It consists of 10 items with a total score ranging from 12 to 66; a higher score indicates a higher level of social support. A score of SSRS ≤22 is considered poor social support, one between 23 and 44 is moderate social support, and one between 45 and 66 scores indicates adequate social support. There are three dimensions included: objective support, subjective support, and support utilization. Objective support reflects on individual social networks and whether they have received instrumental or emotional support in the past. Subjective support represents individual subjective perceptions, such as the emotional experience of being respected, supported, and understood. Support utilization explains how to seek and use social support [23]. Since its establishment in 1986 by Xiao Shuiyuan, the SSRS has been applied in many studies in China. The predictive validity of SSRS is high. To ensure the validity of the questionnaire contents, items that matched the purpose of this study were carefully selected from past studies. The reliability of the questionnaire was evaluated by pretesting it on 50 elderly people aged 60 years or older. The retest coefficients of the second and third sections were 0.800, 0.89–0.94, respectively, while the Cronbach’s α coefficients were 0.934 and 0.920, respectively.

### Analysis procedures

Statistical analyses were performed with SPSS software (Statistical Product and Service Solutions, SPSS: An IBM Company, version 22.0, IBM Corporation, Armonk, NY, USA). Sociodemographic characteristics and responses to each question were described using frequencies and percentages. Spearman correlative analysis was conducted to assess the correlation between dimensions of mutual-support needs and the correlation between mutual-support willingness (unwilling, willing) and needs (low, moderate, high). The Chi-square test was used to examine associations between willingness to receive mutual-support and individual characteristics, social support, and mutual-support needs. Factors associated with mutual-support or mutual-support needs were identified by using multiple logistic regression, which included variables that were statistically significant at the nominal two-sided *P* < 0.05 level in the above univariate analyses. The odds ratio (*OR*) and 95% confidence interval (*CI*) were used to quantify associations.

## Results

The electronic questionnaire was agreed by the participants first and then filled in, and 2167 were returned. The questionnaires were all completed, but some were incompletely filled out, so 65 questionnaires were excluded from the study. As a result, 2102 (97%) valid questionnaires were analyzed.

### Characteristics of the participants and descriptive statistics of each variable

Participants’ characteristics appear in Tables 1 and 2 of the characteristics of the participants, 1243 (59.1%) were aged between 60 and 70 years, and 626 (29.8) were aged between 70 and 80 years, while 233 (11.1) were aged over 80 years. And the overall sample contained more women (57.2%) than men. The majority of the respondents had only primary or no formal education. In addition, 72.7% of participants were married, and 52.4% could take care of themselves. Concerning the nation, 88% of participants belong to the Han nationality. Other characteristics of the participants’ details can be found in Table 1. Participants were willing to participate in mutual-support after understanding the mutual-support model, which was 86.8%. Most of them took a positive attitude towards mutual support. The mean score on the demand for mutual-support for the aged was 66.37 ± 14.396, which means that there is a mutual-support demand for the elderly in rural areas. The score on the SSRS (social support rating scale) was 36.944 ± 6.487, and most of the participants (86.4%) were in moderate social support (see Table 3).

**Table 1:** Univariate analysis of Chi-square test about mutual-support willingness

Characteristic	*n* = 2102(%)	be willing *n* (%)	*P*-value
**Individual characteristic**			
Age (years)			
60–70	1243 (59.1)	1089 (87.6)	0.417
70–80	626 (29.8)	535 (85.5)	
80~	233 (11.1)	201 (86.3)	
Sex			
Male	899 (42.8)	777 (86.4)	0.346
Female	1203 (57.2)	1048 (87.1)	
nation			
Minority nationality	252 (12.0)	202 (80.2)	0.001
Han nationality	1850 (88.0)	1623 (87.7)	
Education level			
Illiteracy	375 (17.8)	325 (86.7)	0.600
primary school	921 (43.8)	807 (87.6)	
Secondary and higher	806 (38.3)	693 (86.0)	
marital status			
Never married	89 (4.2)	76 (85.4)	<0.001
Married	1528 (72.7)	1354 (88.6)	
Divorced	63 (3.0)	34 (54.0)	
Widowed	422 (20.1)	361 (85.5)	
The physical condition			
can’t take care of oneself	54 (2.6)	38 (70.4)	<0.001
Partially self-care	863 (41.1)	728 (84.4)	
Can take care of oneself	1185 (56.4)	1059 (89.4)	
**Family environment**			
Number of children			
0	42 (2.0)	36 (85.7)	<0.001
1	298 (14.2)	237 (79.5)	
2 or more	1762 (83.8)	1552 (88.1)	
Who cares for life			
Oneself	707 (33.6)	628 (88.8)	<0.001
spouse	938 (44.6)	850 (90.6)	
children	384 (18.3)	299 (77.9)	
Others	73 (3.5)	63 (65.8)	
Relationships among family members			
Not harmonious	132 (6.3)	72 (54.5)	<0.001
General harmony	482 (22.9)	397 (82.4)	
harmonious	1488 (70.8)	1356 (91.1)	
Does the family support mutual support			
not support	222 (10.6)	126 (56.8)	<0.001
General support	820 (39.0)	719 (87.7)	
support	1060 (50.4)	980 (92.5)	
**Economic status**			
Average monthly income			
Less than 200 yuan	975 (46.4)	845 (86.7)	0.866
200 to 500 yuan	454 (21.6)	392 (86.3)	
Over 500 yuan	673 (32.0)	588 (87.4)	
source of income			
Subsistence allowances or pension	742 (35.3)	630 (84.9)	0.003
Income from labor	457 (21.7)	413 (90.4)	
From children	697 (33.2)	604 (86.7)	
Other sources	206 (9.8)	178 (86.4)	
Satisfaction with the current economic situation			
No	339 (16.1)	244 (72.0)	<0.001
General	956 (45.5)	861 (90.1)	
Yes	807 (38.4)	720 (89.2)	
**Medical factors**			
Self-assessment of health status			
Unhealthy	392 (18.6)	305 (77.8)	<0.001
Suboptimal	889 (42.3)	788 (88.6)	
healthy	821 (39.1)	732 (89.2)	
Current diseases			
No disease	731 (34.8)	652 (89.2)	0.049
One or two disease	1092 (52.0)	938 (85.9)	
Three or more	279 (13.3)	235 (84.2)	
Regular physical examination			
No	1287 (61.2)	1134 (88.1)	0.029
Yes	815 (38.8)	691 (84.8)	
Health knowledge needs			
No	282 (13.4)	183 (64.9)	<0.001
General	934 (44.4)	837 (89.6)	
Yes	886 (42.2)	805 (90.9)	
Medical accessibility (Whether serious illness can be treated in time)			
No	573 (27.3)	459 (80.1)	<0.001
Yes	1529 (72.7)	1366 (89.3)	
**mutual-support behavior**			
government support			
No	535 (25.5)	398 (74.4)	<0.001
Yes	1567 (74.5)	1427 (91.1)	
Accepting others support			
No	261 (12.4)	147 (56.3)	<0.001
General	955 (45.4)	861 (90.2)	
Yes	886 (42.2)	817 (92.2)	
help others			
No	197 (9.4)	85 (43.1)	<0.001
General	937 (44.6)	837 (89.3)	
Yes	968 (46.1)	903 (93.3)	
**Mutual-support needs**			
Low	542 (25.8)	440 (81.2)	<0.001
moderate	1210 (57.6)	1082 (89.4)	
high	350 (16.7)	303 (86.6)	
**Social support**			
Low	32 (1.5)	17 (53.1)	<0.001
moderate	1815 (86.3)	1567 (86.3)	
high	255 (12.1)	241 (94.5)	
**participate in mutual support after understanding**			
No	277 (13.2)		
Yes	1825 (86.8)

**Table 2:** Univariate analysis of the Chi-square test about mutual-support needs

Characteristic	*n* = 2102(%)	moderate /high n (%)	*P*-value
**Individual characteristic**			
Age (years)			
60–70	1243 (59.1)	883 (71.0)	<0.001
70–80	626 (29.8)	480 (76.7)	
80~	233 (11.1)	197 (84.5)	
Sex			
Male	899 (42.8)	644 (71.6)	0.033
Female	1203 (57.2)	916 (76.1)	
nation			
Minority nationality	252 (12.0)	195 (77.4)	0.393
Han nationality	1850 (88.0)	1365 (73.8)	
Education level			
Illiteracy	375 (17.8)	297 (79.2)	<0.001
primary school	921 (43.8)	687 (74.6)	
Secondary and higher	806 (38.3)	576 (71.5)	
marital status			
Never married	89 (4.2)	62 (69.7)	<0.001
Married	1528 (72.7)	1114 (72.9)	
Divorced	63 (3.0)	47 (74.6)	
Widowed	422 (20.1)	337 (79.9)	
The physical condition			
can’t take care of oneself	54 (2.6)	44 (81.5)	<0.001
Partially self-care	863 (41.1)	713 (82.6)	
Can take care of oneself	1185 (56.4)	1059 (89.4)	
**Family environment**			
Number of children			
0	42 (2.0)	22 (52.3)	<0.001
1	298 (14.2)	228 (76.5)	
2 or more	1762 (83.8)	1310 (74.3)	
Who cares for life			
Oneself	707 (33.6)	499 (70.6)	<0.001
spouse	938 (44.6)	696 (74.2)	
children	384 (18.3)	299 (77.9)	
Others	73 (3.5)	48 (65.8)	
Relationships among family members			
Not harmonious	132 (6.3)	90 (68.2)	0.001
General harmony	482 (22.9)	389 (80.7)	
harmonious	1488 (70.8)	1081 (72.6)	
Does the family support mutual support			
not support	222 (10.6)	157 (70.7)	0.062
General support	820 (39.0)	602 (73.4)	
support	1060 (50.4)	801 (75.6)	
**Economic status**			
Average monthly income			
Less than 200 yuan	975 (46.4)	730 (74.9)	0.125
200 to 500 yuan	454 (21.6)	345 (76.0)	
Over 500 yuan	673 (32.0)	485 (72.1)	
source of income			
Subsistence allowances or pension	742 (35.3)	560 (75.5)	0.006
Income from labor	457 (21.7)	324 (70.9)	
From children	697 (33.2)	526 (75.5)	
Other sources	206 (9.8)	150 (72.8)	
Satisfaction with the current economic situation			
No	339 (16.1)	251 (74.0)	<0.001
General	956 (45.5)	750 (78.5)	
Yes	807 (38.4)	559 (69.3)	
**Medical factors**			
Self-assessment of health status			
Unhealthy	392 (18.6)	316 (80.6)	<0.001
Suboptimal	889 (42.3)	692 (77.8)	
healthy	821 (39.1)	552 (67.2)	
Current diseases			
No disease	731 (34.8)	491 (67.2)	0.049
One or two disease	1092 (52.0)	840 (76.9)	
Three or more	279 (13.3)	229 (82.1)	
Regular physical examination			
No	1287 (61.2)	971 (75.4)	0.142
Yes	815 (38.8)	589 (72.3)	
Health knowledge needs			
No	282 (13.4)	182 (64.5)	<0.001
General	934 (44.4)	666 (71.3)	
Yes	886 (42.2)	712 (80.4)	
Medical accessibility (Whether serious illness can be treated in time)			
No	573 (27.3)	469 (81.8)	<0.001
Yes	1529 (72.7)	1091 (71.4)	
**mutual-support behavior**			
government support			
No	535 (25.5)	397 (74.2)	<0.001
Yes	1567 (74.5)	1163 (74.2)	
Accepting others support			
No	261 (12.4)	162 (62.1)	<0.001
General	955 (45.4)	700 (73.3)	
Yes	886 (42.2)	698 (78.8)	
help others			
No	197 (9.4)	143 (72.6)	<0.001
General	937 (44.6)	677 (72.3)	
Yes	968 (46.1)	740 (76.4)	
**Social support**			
Low	32 (1.5)	16 (50.0)	0.006
moderate	1815 (86.3)	1351 (74.4)	
high	255 (12.1)	193 (75.7)	
**participate in mutual support after understanding**			
No	277 (13.2)	175 (63.2)	<0.001
Yes	1825 (86.8)	1385 (75.9)	
**Mutual-support needs**			
Low	542 (25.8)		
moderate	1210 (57.6)		
High	350 (16.7)

**Table 3:** Social support rating scale (SSRS)

Category	***N*** Ratio (%)	Willing to mutual support	Score (Mean ± SD)	***P***
Objective support			9.066 ± 2.783	
Subjective support			20.258 ± 3.954	
Support utilization			7.579 ± 2.019	
Social support			36.944 ± 6.487	<0.001
Low (≤22)	32 (1.5)	17 (53.1)		
Moderate (22–44)	1815 (86.4)	1567 (86.3)		
High (>45)	255 (12.1)	241 (94.5)		

### Factors influencing mutual-support willingness among the rural elderly

Participants were willing to participate in mutual-support after understanding the mutual-support model, which was 86.8%. Most of them took a positive attitude towards mutual-support. Using the Chi-square test to select meaningful independent variables to enter into the regression model. The results showed that mutual-support willingness differed significantly by individual characteristics (nation, marital status, physical condition), family environment (number of children, who cares for life, relationships among family members, family support), economic status (source of income, satisfaction with the current economic situation), medical factors (self-assessment of health status, current diseases, regular physical examination, health knowledge needs, medical accessibility), and mutual-support behavior (government support, accepting others’ support, helping others). Logistic regression was conducted to determine the factors affecting mutual-support willingness. Finally, there are eleven variables in the regression model equation: marital status, who cares for life, satisfaction with the current economy, relationships among family members, family support, regular physical examination, government support, accepting others support, helping others, mutual-support needs, and social support were related to mutual-support willingness among the rural elderly (see Table 4). The results show that the willingness of mutual-support participation was higher for older people who had harmonious relationships with family members, had family support or government support, and were willing to help others or accept others’ support. And marital status, who cares for life, satisfaction with the current economy, and the regular physical examination also influence participants’ mutual-support willingness. The willingness to mutual-support was higher among persons who are married (*OR* = 2.353, 95% *CI* = [1.019–5.436], *p* < 0.05) compared with those unmarried. People who cared for their spouse (*OR* = 2.955, 95% *CI* = [1.532–5.699], *p* < 0.05) or Children (*OR* = 3.051, 95% *CI* = [1.565–5.946], *p* < 0.05) were more willing to offer mutual support than those who cared for themselves. The mutual-support willingness of participants who had regular physical examinations (*OR* = 1.382, 95% *CI* = [1.008–1.894], *p* < 0.05) is 1.382 times greater than without physical examination. Further, the willingness to mutual-support was positively correlated with mutual support needs and social support (see Table 5).

**Table 4:** Multivariate analysis of factors associated with the mutual-support willingness (*n* = 2102)

Variable	Unwilling n (%)	be willing n (%)	***P***-value	***OR***	95% ***CI***
**marital status**			**0.004**	**0.769**	**0.643–0.921**
single	13 (14.6)	76 (85.4)		1	
married	174 (11.4)	1354 (88.6)	0.045	2.353	1.019–5.436
Divorce	29 (46.0)	34 (54.0)	0.084	1.428	0.954–2.138
Widowed	61 (14.5)	361 (85.5)	0.550	0.802	0.390–1.651
**Who cares for life**			**<0.001**	**0.687**	**0.575–0.821**
oneself	79 (11.2)	628 (88.8)		1	
spouse	88 (9.4)	850 (90.6)	0.001	2.955	1.532–5.699
Children	85 (22.1)	299 (77.9)	0.001	3.051	1.565–5.946
Others	25 (34.2)	48 (65.8)	0.239	1.492	0.766–2.904
**Satisfaction with the current economic**			**<0.001**	**1.178**	**1.029–1.479**
No	95 (28.0)	244 (72.0)		1	
General	95 (9.9)	861 (90.1)	0.005	0.525	0.334–0.826
Yes	87 (10.8)	720 (89.2)	0.002	0.824	0.834–0.979
**Relationships among family members**			**0.008**	**1.401**	**1.092–1.796**
Not harmonious	60 (45.5)	72 (54.5)		1	
General	85 (17.6)	397 (82.4)	0.046	0.590	0.351–0.990
harmonious	132 (8.9)	1356 (91.1)	0.023	0.753	0.525–0.980
**family support**			**<0.001**	**1.743**	**1.400**–**2.17**
not support	96 (43.2)	126 (56.8)		1	
General	101 (12.3)	719 (87.7)	<0.001	0.270	0.175–0.415
Support	80 (7.5)	980 (92.5)	0.027	0.737	0.514–0.778
**Regular physical examination**			**0.014**	**0.670**	**0.487**–**0.921**
No	153 (57.5)	113 (42.5)		1	
Yes	124 (15.2)	691 (84.8)	0.045	1.382	1.008–1.894
**government support**			**<0.001**	**2.033**	**1.484–2.784**
No	137 (40.9)	198 (59.1)	<0.001	1	0.335–0.634
Yes	140 (8.9)	1427 (91.1)		0.461	
**Accepting Others support**			**<0.001**	**1.549**	**1.221–1.965**
No	114 (43.7)	147 (56.3)		1	
General	94 (9.8)	861 (91.2)	<0.001	0.398	0.255–0.619
Yes	69 (7.8)	817 (92.2)	0.572	1.123	0.751–1.679
**help others**			**<0.001**	**2.089**	**1.611–2.708**
No	112 (56.9)	85 (43.1)	<0.001	1	
General	100 (10.7)	837 (90.3)	0.334	0.165	0.102–0.266
Yes	65 (6.7)	903 (93.3)		0.822	0.552–1.223
**mutual support needs**			**0.007**	**1.399**	**1.098**–**1.783**
low	102 (18.8)	440 (81.2)	0.006	1	
moderate	128 (10.6)	1082 (89.4)	0.039	0.520	0.327–0.827
high	47 (13.4)	303 (86.6)		0.918	0.728–0.987
**Social support**			**0.017**	**1.594**	**1.096–2.803**
low	15 (46.9)	17 (53.1)		1	
moderate	248 (13.7)	1567 (86.3)	0.005	0.192	0.061–0.604
high	14 (5.5)	241 (94.5)	0.032	0.731	0.394–0.857

**Table 5:** Correlation analysis between different dimensions of elderly mutual-support

		2	3	4	mutual-support needs	Social support
1. Daily life care	1	0.494***	0.496***	0.375***		
2. Health service	0.494***	1	0.672***	0.542***		
3. Spiritual comfort	0.496***	0.672***	1	0.760***		
4. Entertainment and learning	0.375***	0.542***	0.760***	1		
5. mutual-support willingness					0.068**	0.118***

Report Spearman’s correlation coefficients

*p < .05. **p < .01. ***p < .001

### Factors influencing mutual-support needs among the rural elderly

Table 5 shows the correlation of mutual-support needs across four dimensions. Each dimension was significantly correlated. Table 6 shows the scores of mutual-support for the rural elderly. The total average score of the rural elderly’s demand for mutual-support was 66.37 ± 14.396. The daily life care service needs scores were the lowest among the four dimensions, at 14.620 ± 6.143. And with the health service needs score being the highest, which was 17.390 ± 1.080. The elderly in this study scored relatively higher on health service needs, probably due to the shortage of medical and health resources in rural areas. Regarding items in spiritual comfort service, entertainment, and learning, subjects also have demands on their lives. Using the Chi-square test to select meaningful independent variables to enter into the regression model. The results showed that mutual-support needs differed significantly by individual characteristic (age, sex, education level, marital status, physical condition), family environment (number of children, who cares for life, relationships among family members), economic status (source of income, satisfaction with the current economic situation), medical factors (self-assessment of health status, current diseases, regular physical examination, health knowledge needs, medical accessibility), and mutual-support behavior (government support, accept others support, help others). Logistic regression was conducted to determine the factors affecting mutual-support needs. Finally, there are seven variables in the regression model equation. Age, sex, physical condition, health knowledge needs, medical accessibility, and so on were related to mutual-support needs among the rural elderly (see Table 7). These show that participants who were older, male, had poor self-care ability, serious illness that could not be treated in time, and were willing to accept other people’s help had higher mutual-support needs. The need for mutual support was higher among people who are aged more than 80 years old (*OR* = 0.631, 95% *CI* = [0.418–0.955], *p* < 0.05) compared with those aged 60 to 70 years old. The need of mutual-support was lower among people who are partially self-care (*OR* = 2.097, 95% *CI* = [1.008–4.365], *p* < 0.05) or can take care of themselves (*OR* = 2.224, 95% *CI* = [1.767–2.800], *p* < 0.05) compared with those who can’t take care of themselves. The mutual-support needs for participants who were female (*OR* = 0.771, 95% *CI* = [0.627–0.947], *p* < 0.05) is 0.771 times greater than those of males (see Table 7).

**Table 6:** Demand score of mutual-support for the elderly

Project	Item score	score (Mean ± SD)
**Daily life care service needs**		**14.620 ± 6.143**
1. Purchasing daily necessities	5.000	2.80 ± 1.103
2. Physical work	5.000	3.10 ± 1.117
3. Washing and cooking	5.000	2.77 ± 1.101
4. Cleaning	5.000	2.79 ± 1.106
5. Agency service	5.000	3.16 ± 1.158
**Health service needs**		**17.390 ± 1.080**
1. Escort to the hospital when sick	5.000	3.16 ± 1.158
2. Take care of by others when sick	5.000	3.59 ± 1.036
3. Help to measure blood pressure or blood sugar regularly	5.000	3.69 ± 0.963
4. Remind to take medicine	5.000	3.44 ± 1.013
5. Health knowledge popularization	5.000	3.51 ± 0.943
**Spiritual comfort service needs**		**16.370 ± 3.987**
1. Accompany and chat	5.000	3.51 ± 0.978
2. Participate in social activities	5.000	3.27 ± 0.987
3. psychological counseling	5.000	2.92 ± 1.049
4. Telephone care and greetings	5.000	3.36 ± 1.024
5. Accompany out for a walk	5.000	3.25 ± 1.022
**Entertainment and learning needs**		**15.930 ± 3.915**
1. Entertainment (chess, cards, etc.)	5.000	3.26 ± 1.045
2. Accompany to exercise	5.000	3.18 ± 1.024
3. Accompany to watch TV	5.000	3.12 ± 1.108
4. Attending lectures on health knowledge for the elderly	5.000	3.15 ± 0.995
5. Learning new things	5.000	3.31 ± 0.953
**Demand score of mutual support**		**66.37 ± 14.396**

**Table 7:** Multivariate analysis of factors associated with mutual-support needs (*n* = 2102)

Variable	Low ***n*** (%)	moderate /high ***n*** (%)	***P***-value	***OR***	95% ***CI***
**Age**			**0.011**	**1.250**	**1.053–1.484**
60–70	360 (29.0)	883 (71.0)		1	
70–80	146 (23.3)	480 (76.7)	0.002	0.531	0.359–0.783
80~	36 (15.5)	197 (84.5)	0.029	0.631	0.418–0.955
**Sex**			**0.036**	**1.254**	**1.014–1.550**
Male	255 (28.4)	644 (71.6)		1	
Female	287 (23.9)	916 (76.1)	0.013	0.771	0.627–0.947
**The physical condition**			**<0.001**	**0.622**	**0.490–0.790**
can’t take care of oneself	10 (18.5)	44 (81.5)		1	
Partially self-care	150 (17.4)	713 (82.6)	0.048	2.097	1.008–4.365
Can take care of oneself	382 (32.2)	803 (67.8)	<0.001	2.224	1.767–2.800
**Health knowledge needs**			**<0.001**	**1.646**	**1.389–1.952**
No	100 (35.5)	182 (64.5)		1	
general	268 (28.7)	666 (71.3)	<0.001	0.442	0.319–0.612
Yes	174 (19.6)	712 (70.4)	<0.001	0.608	0.483–0.766
**Medical accessibility (Whether serious illness can be treated in time)**			**0.002**	**0.639**	**0.481–0.850**
No	104 (18.2)	469 (81.8)		1	
Yes	438 (28.6)	1091 (71.4)	<0.001	1.782	1.376–2.306
**Accepting others support**			**<0.001**	**1.397**	**1.168–1.671**
No	99 (38.0)	162 (62.0)		1	
General	255 (26.7)	700 (73.3)	<0.001	0.534	0.383–0.745
Yes	188 (21.2)	698 (70.8)	0.046	0.792	0.630–0.996
**participate in mutual support after understanding**			**<0.001**	**1.964**	**1.419–2.718**
No	102 (36.8)	175 (62.2)		1	
Yes	440 (24.1)	1385 (75.9)	<0.001	0.597	0.439–0.814

### Social support affects mutual-support willingness and needs among the elderly in rural areas

The total score on social support for the aged was 36.944 ± 6.487, with 86.4% at a moderate level. The subjective support score was relatively high. Social support affects participants’ mutual-support willingness. And the results show that the higher the degree of social support, the more inclined people are to participate in mutual-support (see Table 3).

## Discussion

This study contributes to a better understanding of the mutual-support willingness between mutual-support needs and social support. Most of the rural elderly hold a positive attitude towards mutual-support after understanding it. The higher the degree of social support, the stronger the willingness of the rural elderly to participate in mutual-support. The rural elderly with mutual-support needs were more likely to take part in mutual-support.

### Mutual-support willingness

The first research question addressed the willingness of mutual-support. Participants were willing to participate in mutual support after understanding the mutual support model, at 86.8%, and this was significantly higher compared with the rate among Japanese (57.8%) [15]. One of the reasons for this difference could be the rural elderly’s traditional culture of helping each other in China is more aware of the significance of mutual-support [24]. In this study, as for individual characteristic factors, there is no significant relationship between the willingness to participate in mutual support and the gender or age of the elderly. And the willingness to participate in mutual support was related to marital status, who cares for life and physical condition. From the perspective of age, most scholars believe that with the growth of age, the physical strength and intelligence of the elderly gradually decline, which will lead to a decrease in their willingness to participate in mutual-support [25]. From the perspective of educational level, scholars generally believe that with the continuous improvement of the educational level of the elderly, their willingness to participate in mutual-support is higher [26]. The elderly with good health were more willing to participate in mutual-support. The domestic multi mathematician’s research shows that good health is the necessary condition for the elderly to help others [27]. But, Xu Jiaming, in the study of specific groups of the elderly, found no significant relationship between the willingness to participate in mutual-support and the gender, age, and educational background of the elderly [28], which was the same as our study. And one of the important factors was marital status. The existence of spouses plays a necessary role in emotional support and stress relief for the elderly, making them more inclined to provide mutual assistance services [26]. As for family security factors, relationships among family members and family support were significant with mutual-support willingness. This result indicates that family members have an important influence on the decision of the elderly to participate in mutual-support [29]. One study found that, as a form of intergenerational relations, support from offspring would directly or indirectly affect the older adults’ long-term care willingness [30]. Rural family structure, lifestyle, cultural foundation, and emotional foundation make it possible to help each other and support each other, which has become a strong support for the operation of the mutual support model. As for economic security factors, there is no significant relationship between the willingness to participate in mutual-support and the average monthly income of the elderly. And the willingness to participate in mutual-support was related to the source of income and satisfaction with the current economic situation. In China, due to traditional norms of child rearing and caring for the elderly [31], many elderly people expect to be financially supported by their children [32]. It’s expectable that children will financially support their elderly parents. This will be even greater in rural areas, where there are lower levels of state financial investment and support [33]. In this study, the economy was relatively lower than those reported in urban areas, indicating double influence among the rural elderly regarding mutual-support willingness. First, economic conditions are one of the important factors that restrict the mutual-support participation of the elderly. Only by ensuring that they have no financial worries and that the elderly participate in community mutual-support for the elderly, can they have no worries. But for the elderly without income, they may be more inclined to get help, and their willingness to mutual-support may be stronger. As for medical security factors, health status and medical accessibility have a significant impact on the decision of the elderly to participate in mutual-support. Health care and medical care are the priority for eldercare, which is consistent with previous research findings [34, 35]. Most rural elderly people have no regular physical examination habits and find it inconvenient to go to the hospital. That means, due to economic and environmental constraints, most of the rural elderly have fewer medical resources, and lots of them have no children around because of the transfer of young and middle-aged rural labor, so medical security was an important factor in influencing the mutual-support willingness. Finally, we clarified the influencing factors of the rural elderly’s willingness to participate in mutual-support and gave them adequate protection and care to ease their worries about participating in mutual-support.

### Mutual-support needs

Addressing the unmet care and support needs of an aging population, and designing services and solutions centered around what older people need or want, is becoming an urgent public health priority [36]. Addressing these unmet needs is becoming one of the most urgent public health priorities. To develop effective solutions to address some of these needs, it is important first to understand the care and support needs of older people. The total average score of the rural elderly’s demand for mutual-support is at a relatively high level. And the demand for health services, spiritual comfort services, and entertainment and learning needs is slightly higher than the demand for daily life-care services. The highest score was health service demand, which is consistent with the results of relevant studies [37, 38], possibly due to the risk of disease. With increasing age, the physical function of the elderly will degenerate and become more and more unpredictable. In recent years, more and more diseases will follow, and the demand for health services will be stronger. In terms of health service demand, the elderly in rural areas have the highest demand for medical escort and care when they are sick. Due to the inconvenient transportation, low economic level, and insufficient primary health resources in rural areas, it is more difficult for the elderly to seek medical treatment, so the demand for medical escort is higher. Moreover, the long-term absence of the children of the empty nest elderly will inevitably affect the availability of their care resources. Even when they are sick, their children can’t continue to care for them, and their physical condition is poor. The influence on the elderly is particularly prominent. It has been proven that daily care services provided by the community may liberate older adults from burdensome housework and allow them to spare more time for exercise and relaxation, which might be beneficial for their physical and mental health [39, 40]. For daily life care service needs, it is lower than others. Perhaps decisions in China took family resources and cultural norms into account, and many elderly people did not require assistance with daily activities [41, 42]. Our findings suggest a significant demand for spiritual comfort services for the elderly. Traditionally, the family is the main source of spiritual and emotional support for the Chinese elderly [43]. However, as the traditional intergenerational co-residence mode has been decreasing and the traditional perception has been eroding in the past two decades, other care services have been emerging as a supplement to family support [44]. In particular, spiritual comfort services like psychological counseling and the company of volunteers, provide older adults with professional psychological services and work as a new way to help them regain emotional and social support, which may possibly make them feel supported and secure, and further improve their mutual-support willingness [45]. Recreational services in China provide leisure activities appropriate to older adults in some elderly activity centers for entertainment and learning needs, which have been proven to be an effective intervention for the improvement of the elderly’s health and life satisfaction [46]. However, the lack of exercise and entertainment facilities in rural areas remains an important issue.

To improve the health status and meet the mutual-support needs of the rural elderly, more financial support and human resources are expected to be invented in aspects of expanding mutual-support service coverage and improving the accessibility of these services for the elderly in China, especially in rural areas and economically undeveloped areas. Furthermore, we must pay greater attention to the needs for health care and spiritual comfort services.

### Social support

The effect of social support on the health of the elderly has been the focus of research. In the United States, social support for the elderly has been shown to prevent further deterioration of their health [44]. Based on assessing a large amount of research, it is shown that supportive relationships protect us from a multitude of mental health problems [47]. The total score for social support for the aged was 36.944 ± 6.487, at a moderate level. And the higher the degree of social support, the stronger the willingness to participate in mutual-support for the aged. Rosow held that the effectiveness of social support for the rural elderly decreased among children, neighbors, and friends, and when the rural elderly fell ill, their neighbors would provide the most essential social support if their children were not around [19]. A study from South Korea showed that social support from significant others and friends was significantly associated with the elderly’s health-related quality [48]. Walker and Hiller found that physical health and subjective well-being could be directly affected by perceptions of neighborhood relationships in the community. In other words, establishing a social support network in the community through reciprocal trust can not only make them feel more supported but also improve their life satisfaction [49]. The rural elderly shared their experiences and knowledge and provided each other with necessary help through life or emotional support at the mutual-management stage, thereby developing new relationships to add to the support network, which also contributed to the significant increases in their SSRS scores. Take measures to improve social support for the elderly in rural areas, to improve the happiness of the elderly in their later years, and to promote the development of rural eldercare to alleviate the crisis of population aging.

### Limitations

Our study had several limitations. First, the generalizability of our data to other countries’ elderly may be limited because the sample was from one province of China only. More studies are needed to widen the sample selection and include more potential factors affecting the elderly’s willingness to choose mutual-support, especially those factors for which specific interventions can be valued and solved by society and the government, such as rural endowment insurance. However, the results of this study have valuable implications for the aged about the factors influencing mutual-support willingness and needs in China and other developing countries. Second, sociocultural factors possibly related to mutual-support willingness and mutual-support needs, such as beliefs and tradition, were not explored, because only a quantitative method was used.

## Conclusion

In conclusion, the elderly population in rural areas of China generally lack knowledge about mutual-support model. Most of the participants adopted a positive attitude towards help from and helping others after understanding the mutual support model. It is necessary to objectively evaluate the factors related to mutual-support willingness and needs of the elderly living in rural districts and take steps to enhance social support and meet their demand for mutual-support. Health authorities in China and community health centers need to provide these older people with adequate interventions, such as promoting the rural mutual-support model, encouraging family members or neighbors to provide more support for the elderly, and taking action to improve their care services. This study emphasizes the importance of social support and improved medical, health, and counseling services for the rural elderly, which are of great significance for improving the happiness of the elderly in their later years, promoting the development of rural pensions, and alleviating the crisis of population aging in China.

## Data Availability

The datasets used and/or analysed during the current study are available from the corresponding author on reasonable request. Anonymized participant data used in the preparation of this article will be made available on request from the lead author.
